# Addressing the under-reporting of adverse drug reactions in public health programs controlling HIV/AIDS, Tuberculosis and Malaria: A prospective cohort study

**DOI:** 10.1371/journal.pone.0200810

**Published:** 2018-08-22

**Authors:** Yohanna Kambai Avong, Bolajoko Jatau, Ritmwa Gurumnaan, Nanfwang Danat, James Okuma, Istifanus Usman, Dennis Mordi, Blessing Ukpabi, Gbenga Ayodele Kayode, Saswata Dutt, Osman El-Tayeb, Bamgboye Afolabi, Isah Ambrose, Oche Agbaji, Adeline Osakwe, Ali Ibrahim, Comfort Ogar, Helga Nosiri, Eunice B. Avong, Victor Adekanmbi, Olalekan Uthman, Alash’le Abimiku, Yetunde O. Oni, Charles Olalekan Mensah, Patrick Dakum, Kamau Edward Mberu, Olumide A. T. Ogundahunsi

**Affiliations:** 1 Institute of Human Virology, Nigeria, Maina Court, Central Business District, Abuja, Nigeria; 2 Demian Foundation of Belgium, Ibadan, Oyo State, Nigeria; 3 Health, Environment and Development Foundation, Lagos State, Lagos, Nigeria; 4 Faculty of Pharmaceutical Sciences, University of Benin, Benin City, Edo State, Benin, Nigeria; 5 Jos University Teaching Hospital, Jos, Plateau State, Nigeria; 6 University of Nigeria, Nsukka, Anambra State, Nigeria; 7 National Agency for Food and Drug Administration and Control, Federal Capital Territory, Abuja, Nigeria; 8 Wise Health Services Limited, Wuse, Abuja, Nigeria; 9 Division of Population Medicine, Cardiff University, Cardiff, United Kingdom; 10 Warwick Medical School, University of Warwick, Coventry United Kingdom; 11 Special Program for Research in Tropical Diseases, World Health Organization (TDR), Geneva, Switzerland; Universitat Autonoma de Barcelona, SPAIN

## Abstract

**Background:**

Adverse Drug Reactions (ADRs) are a major clinical and public health problem world-wide. The prompt reporting of suspected ADRs to regulatory authorities to activate drug safety surveillance and regulation appears to be the most pragmatic measure for addressing the problem. This paper evaluated a pharmacovigilance (PV) training model that was designed to improve the reporting of ADRs in public health programs treating the Human Immunodeficiency Virus (HIV), Tuberculosis (TB) and Malaria.

**Methods:**

A Structured Pharmacovigilance and Training Initiative (SPHAR-TI) model based on the World Health Organization accredited Structured Operational Research and Training Initiative (SOR-IT) model was designed and implemented over a period of 12 months. A prospective cohort design was deployed to evaluate the outcomes of the model. The primary outcomes were knowledge gained and Individual Case Safety Reports (ICSR) (completed adverse drug reactions monitoring forms) submitted, while the secondary outcomes were facility based Pharmacovigilance Committees activated and health facility healthcare workers trained by the participants.

**Results:**

Fifty-five (98%) participants were trained and followed up for 12 months. More than three quarter of the participants have never received training on pharmacovigilance prior to the course. Yet, a significant gain in knowledge was observed after the participants completed a comprehensive training for six days. In only seven months, 3000 ICSRs (with 100% completeness) were submitted, 2,937 facility based healthcare workers trained and 46 Pharmacovigilance Committees activated by the participants. Overall, a 273% increase in ICSRs submission to the National Agency for Food and Drug Administration and Control (NAFDAC) was observed.

**Conclusion:**

Participants gained knowledge, which tended to increase the reporting of ADRs. The SPHAR-TI model could be an option for strengthening the continuous reporting of ADRs in public health programs in resource limited settings.

## Background

Adverse Drug Reactions (ADRs) have emerged as a major clinical and public health problem responsible for approximately 5 to 35% of hospital admissions in both developed and developing countries [1–7]. In the United States and Europe, ADRs are among the top ten causes of mortality as well as increasing the cost of care [4, 8, 9 and 10]. In African countries, the introduction of antiretroviral therapy (ART) for the control of HIV/AIDS has led to an upsurge in the cardio-metabolic disorders such as type 2 diabetes mellitus and hypertension. Anti-retroviral drugs–the primary agents in ART, are largely responsible for the rise in cardio-metabolic disorders in sub-Saharan Africa according to some authors [11, 12]. Similarly, the treatment of Drug Resistant Tuberculosis (DRTB) with Second line Anti-tuberculosis drugs (SLDs) is driving the rise of mental illnesses (psychosis), loss of hearing (ototoxicity) and kidney damage in some countries [13].

Prompt reporting of ADRs to drug regulatory bodies is an important drug safety measure but under-reporting is a major challenge even in developed countries with adequate human and material resources to tackle the problem [4, 8, 9 and 10]. A systematic review of 37 studies by Hazell and Shakir found a median under-reporting rate of 94% [14]. Many factors are associated with the under-reporting of ADRs [15]; the commonest factors frequently cited in most of the studies are healthcare workers’ lack of knowledge and poor attitude [16–29]. In a very recent study; Terblanche et al [30] found that 53.8% of the participants gave not “knowing how to report” ADRs as the reason discouraging the reporting. Interestingly, some studies have shown that training could address both the poor attitude and the lack of knowledge leading to increase in the accuracy and rate of reporting of ADRs to regulatory bodies [14, 31].

Prompt reporting is pragmatic and arguably, the best method for drug safety surveillance [32]. The delay in reporting ADRs can be catastrophic; for example, almost seven million patients took Fenfluramine before its association with Valvular Heart Disease (VHD) was reported and the drug withdrawn from the market [33]. Similarly, over 10,000 children in Germany in the early sixties suffered Phocomelia before the causative agent; Thalidomide was identified and withdrawn from clinical practice [34].

Public health programs, especially the HIV/AIDS, Tuberculosis and Malaria have the greatest risk of ADRs because millions of people are treated with a wide range of drugs, some of which have serious/life threatening adverse reactions [35–38]. In the western countries where ARVs have been used for many years, cases of rising obesity, weight gain and cardio-metabolic diseases are persistently being reported in association with the use of ARVs [39–40]. The demand for drug safety surveillance has therefore become a major consideration in the global scale up of ART to end HIV/AIDS and TB. For instance, the World Health Organization (WHO) requires countries adopting the “shorter regimen” in the control of DRTB to institute active drug safety monitoring (aDSM).

### The pharmacovigilance system in Nigeria

Pharmacovigilance systems (PVS) refer to schemes that are established to facilitate the reporting of suspected ADRs to national or international bodies responsible for the monitoring of drug safety and regulations. Countries participating in the international drug monitoring scheme are required by regulation to collect and submit their reports to the International Drug Monitoring Center in Geneva.

Nigeria joined the International Drug Monitoring Scheme in 2004 and became the 74^th^ member country. The National Agency for Food and Drug Administration and Control (NAFDAC)–the body responsible for drug safety and regulation in Nigeria, thereafter, developed a National Pharmacovigilance Policy and instituted an administrative structure, consisting of the National Pharmacovigilance Center (NPC) in Abuja and Zonal Pharmacovigilance Centers (ZPC) in each of the six geopolitical regions of the country.

NAFDAC also instituted the National Pharmacovigilance System which involves signal detection, collection, collation and analysis of ADRs [41]. Organizations or individuals holding a marketing authorization for marketing medicinal products are mandated to report any suspected ADR associated with the product they are authorized to market [41]. Healthcare providers (pharmacists, doctors and nurses) are also required by the government to report suspected ADRs, although this is not mandatory [41] as is the case in Sweden, France and Italy [42].

The primary tool for reporting ADRs in Nigeria is a structured in-take form known as the “Adverse Drug Reactions Form” (ADR Form) (Figure A in S1 File) available at https://www.nafdac.gov.ng. This form is similar to the United Kingdom’s Yellow Card and has five major sections, which must all be accurately completed. A fully completed ADR form is known as the “Individual Case Safety Report” (ICSR).

The quality of an ICSR is directly proportional to the amount of clinically relevant information that is included [43–46]. On this basis, an ICSR with 100% completeness is expected to have the highest quality provided that the information included in all the sections is accurate. In Nigeria, poor quality ICSRs are usually quarantined by the NPC as they cannot be sent to the International Drug Monitoring Center in Uppsala, Sweden.

A typical ICSR provides the following information:

- Patient details (name, age, sex and weight)
- Adverse drug reaction (description, date reaction started and stopped and outcome–recovered fully, congenital abnormality, recovered with abnormality, life threatening and death)
- Suspected drug (brand and generic names, batch number, NAFDAC number, expiry date)
- Concomitant medicines (all medicines taken in the last three months)
- Source of report

The reporting of ADRs to NAFDAC follows two major steps:

- Accurate completion of the ADR forms when a suspected ADR is observed by healthcare providers or reported by a patient during routine treatment of health conditions. Marketing Authorization Holders (MAH) are also expected to complete the ADR forms when suspected ADR are reported to them by patients, health institutions or the healthcare workers within and outside the country.
- Dispatch the ICSRs to the NPC in Abuja.

The ICSRs are dispatched to the NPC in Abuja through different means; the frequently used method is surface mail posting of the ICSRs to the NPC by the health facilities or visiting the health facilities by the NAFDAC designated staff to pick up the ICSRs. ICSRs could also be scanned and sent as “attachment” through emails communication. The NPC validate and analyze the submitted ICSRs and extract the relevant information into VigiBase–a proprietary web database (https://www.who-umc.org/vigibase/vigibase/) hosted at the WHO collaborating center for international drug monitoring in Uppsala, Sweden.

### The pervasive problem of under-reporting of adverse drug reactions in Nigeria

The under-reporting of ADRs in Nigeria has been documented in several studies [47–50]. According to NAFDAC, only 16,500 ICSRs out of 80,000 ADR Forms distributed nation-wide for 12 years (2004 to 2016), were submitted back to NAFDAC [51]. This is equivalent to submitting only 1,375 ICSRs per year. The WHO criteria for adequate reporting of ADRs are 200 reports per million inhabitants per year [52]. With a population of 170 million inhabitants in 2016, at least 34,000 ICSRs should have been submitted to NAFDAC instead of the 1,375 ICSRs. Furthermore, Nigeria had 323,941healthcare workers [53–54] (consisting of Physicians, Nurses, Midwives, Pharmacists, Pharmacy-technicians, Radiographers, Medical Laboratory Scientists and Community Health Officers), [55–56] according to the 2005–2007 report of the National Professional Medical/Health Regulatory bodies. In 2015, NAFDAC reported that only 1,385 ICSRs (see Figure B in S1 File) were submitted supposedly by over 323,941 workers. Given the large population of healthcare workers and the overwhelming increase in drug consumption due to the high burden of HIV/AIDS, Tuberculosis and Malaria, submitting only 1,385 ICSRs clearly suggests that Nigeria is facing a major crisis of under-reporting of ADRs. Ironically, the factors that are undermining the reporting of ADRs in other countries are also rife in Nigeria and these include lack of knowledge, inaccurate description of ADRs, poor quality reports and poor compliance to the pharmacovigilance processes (data collection, storage, management, risk assessment and communication) [14,41,44].

The SPHAR-TI model was designed to address the challenge of under-reporting of ADRs in Nigeria through capacity building. Until the SPHAR-TI course, majority (71.0%) of the healthcare workers that participated in the course have never received training in pharmacovigilance but were nonetheless working in public health institutions or hospitals directly treating HIV/AIDS, Tuberculosis and Malaria through the respective public health disease control programs. Some studies have reported high prevalence of ADRs emanating from the HIV/AIDS, TB and Malaria public health programs [13, 35, 37, 38, 39, 40], thus, justifying the SPHARTI model.

### The structured pharmacovigilance and Training Initiative model

The SPHAR-TI model (refer to S2 File) was a 12 month modular course, modelled after the WHO accredited Structured Operational and Training Initiative (SORT-IT) [57,58]. The model incorporated six distinct but inter-related activities, referred to as the SPHAR-TI’s principles. These are: a training workshop; participants’ mobilization; monitoring and evaluating and providing feedback; setting up a reporting system; providing leadership and collaborating with the government. This paper evaluated the model with the main objective of describing the outcomes after the first 12 months of implementation.

## Methods

This manuscript complies with the STROBE reporting standard for observational studies.

### Ethics approval

Ethics approval for the evaluation was given to the Institute of Human Virology Nigeria by the National Health Research Ethics Committee of Nigeria under the title: “Engaging indigenous organization to sustain and enhance clinical services for the prevention, care and treatment of HIV/AIDS in the Federal Republic of Nigeria under the President’s Emergency Plan for AIDS Relief (PEPFAR)”; Number: NHREC/01/01/2007; dated August 12, 2016.

### Setting

The workshop was conducted in the Federal Capital Territory, Abuja but the participants were selected from health facilities and institutions in the six geopolitical regions of Nigeria. Nigeria has six geopolitical regions with a population of 170 million inhabitants. These regions include the North-east, North-west, North-central, South-west, South-east and South-south.

### Study population

The study population consisted of health care workers (Nurses, Physicians and Pharmacists) who were selected for the SPHAR-TI course based on rigorous selection criteria (Table A in S2 File).

### Study design

A prospective cohort design was deployed for the evaluation of the SPHAR-TI model. Participants that attended the SPHAR-TI’s workshop described in S2 File were followed up for 12 months through internet and telephone communication. Performance was evaluated based on meeting defined milestones. Bio-demographic characteristics were recorded and participants’ knowledge assessed prior to the workshop. Five days after the workshop, participants’ knowledge was re-assessed without giving them a prior warning they would be re-assessed. ICSRs were submitted online every three months (31^st^ May, 29^th^ July, 30^th^ September and 30^th^ November 2016). Data on the number of healthcare workers trained by the participants and the Pharmacovigilance Committees activated were submitted online not later than 10^th^ December 2016.

### Outcomes of measure

The aim of the model was to improve the reporting of ADRs from both hospitals based and more importantly, community based public health programs controlling the AIDS epidemic, Tuberculosis and Malaria. The primary outcomes were knowledge gained and the number of ICSRs submitted to NAFDAC. We expected to see a significant gain in knowledge and a remarkable increase in the reporting of ADRs if the model was effective. The secondary outcomes were the health facility staff trained by the participants through the step-down training and the Pharmacovigilance Committees activated.

The secondary outcomes were evaluated because NAFDAC encourages the setting up of Pharmacovigilance Committees in health facilities as a pragmatic strategy for promoting the reporting of ADRs. During the workshop, participants were taught and encouraged to step-down the workshop and activate the committees. We expected the participants to be able to train others and set up new Pharmacovigilance Committees if the model was effective.

### Evaluation of the outcomes of the model

The overall outcome of the model was assessed by comparing the number of ICSRs submitted by the national health workforce in seven months with the number submitted by the participants in seven months. The primary and secondary outcomes were evaluated as described below:

#### Gain in knowledge

Gain in knowledge was assessed using a structured questionnaire, consisting of 24 questions developed by expert physicians and pharmacists in the three diseases (HIV, TB and Malaria). The questions covered the clinical management of HIV/AIDS, Tuberculosis and Malaria and the ADRs associated with the use of antiretroviral, anti-tuberculosis and anti-malaria drugs. The questionnaire also assessed the knowledge of Nigeria’s ADR reporting and pharmacovigilance system.

Prior to the commencement of the workshop, the questionnaire was administered for an hour (pre-test). At the end of the workshop, which was five days after the pre-test, the same questionnaire was re-administered (post-test). To minimize measurement biases, the questionnaire was withdrawn immediately after the pre-test and participants were not warned the questionnaire would be re-administered after the workshop. Participants did not also know the result of the pre-test until after the post-test. The pass mark for the pre and post-tests was 45%.

#### Individual case safety reports submission

The correctness and completeness of the ICSR and the quantities submitted online were assessed. All submitted ICSRs were manually checked for correctness and completeness and the total number of correctly completed forms were counted and recorded in each cycle. The number of ICSRs submitted within seven months were summed up and compared with the amount that would have been submitted by the national health workforce in seven months in 2015.

#### Pharmacovigilance committees activated and training of health facility staff by participants

To assess the two variables, participants were given a spreadsheet for recording their step-down activities after the workshop. The spreadsheet included the following variables:

- Status of the health facility
- Date of training
- Description of training
- Objectives of the training
- Mode of delivery of training content
- Number of doctors, nurses and pharmacists in attendance
- Number of Pharmacovigilance Committees activated
- Involvement of hospital management
- Collaboration with a pharmaceutical company in the training
- Collaboration with NAFDAC.

The completed spreadsheets were submitted to NAFDAC online at different times but not later than 10^th^ December, 2016. We extracted the number of health facility staff trained and the number of Pharmacovigilance Committees’ activated into a template for analysis.

### Statistical analysis

We applied descriptive statistics (mean, percentage and summation) in the analysis of the bio-demographic data and primary and secondary outcomes. The overall outcome of the model was analyzed by calculating the percentage increase in ADR submission using the arithmetic formula: T-M/M*100 [Where T = total number of ICSRs submitted by the participants in seven months; M = total number of ICSR submitted by the national health workforce in seven month]. According to NAFDAC, in 2015, the over 323,941 national health workforce submitted 1,385 ICSRs (refer to Figure B in S1 File), equivalents to 805 ICSRs in seven months. The 3000 ICSRs (T) (submitted by the participants) and 805 ICSRs (M) were plucked into the arithmetic formula to determine the percentage increase in ICSRs submission.

Participants gain in knowledge was analyzed by calculating the difference between the mean pre-test and post-test scores. Summation was applied in analyzing the number of ICSR submitted by the participants, health staff trained by the participants and Pharmacovigilance Committees activated.

## Results

Two hundred and forty seven candidates applied for the course, 56 met the selection criteria and were invited for the workshop but one person could not make it leaving 55 participants, equivalent to 98.2% (55/56) participating rate. Participants’ characteristics are presented in S1 Table. Participants without a previous training in pharmacovigilance were more in number compared with participants that have attended pharmacovigilance training (s) in the past [39(71%) vs 16 (29.1%)].

S2 Table compares the difference in the mean scores (gain in knowledge) among the participants. Participants more than 40 years (9.4 [SD = 7.0]), Pharmacists (8.5 [SD = 7.4]), and Nurses (7.6 [SD = 6.4]), participants from the Roll Back Malaria program (9.3 [SD = 8.8]) and those without previous training (9.5 [SD = 7.8]), appeared to gain knowledge more than participants from other groups.

The outcomes of the model are presented in the S3 Table. Participants demonstrated a significant gain in knowledge (20.4 vs 27.8 (P value < 0.001) and submitted 3000 ICSRs with 100% correctness and completeness. Compared with the 805 ICSRs submitted by more than 323,941 healthcare workers in the general population who were not SPHAR-TI trained, the percentage increase in ICSRs submission was 273%. Participants were also able to independently train 2,937 healthcare workers and activated 46 Pharmacovigilance Committees.

## Discussion

The major finding of this evaluation is the significant gain in knowledge observed among the participants generally. NAFDAC’s concordance on the effectiveness of the model to significantly improve the reporting of ADRs in Nigeria (Figure B in S1 File) buttresses this observation and underscores the potential viability of the model to improve the reporting of ADRs in public health programs. Furthermore, the positive outcome achieved when NAFDAC tested the model in the training of 600 healthcare workers from ten states in Nigeria (Figure B in S1 File), suggests that the model can be replicated in countries facing similar challenge of under-reporting of ADRs with Nigeria.

We also observed four additional outcomes. Firstly, participants developed the capacity to detect and accurately report ADRs including the serious ADRs such as Stevens Johnson Syndrome (SJS) and Bilateral Gynaecomastia (BG). Secondly, the rate of ADR reporting increased by 273%, when compared with the average reporting rate in the general population over the past 12 years. This finding is consistent with the findings from previous studies that examined the impact of training at improving the reporting of ADRs [31, 59]. Thirdly, participants were able to train their peers, thus, they increased the number of healthcare workers for pharmacovigilance service delivery particularly in the communities. In addition, participants developed the capacity to activate Pharmacovigilance Committees in their various health facilities. This is a feat NAFDAC has persistently encouraged in an effort to boost the reporting of ADRs in Nigeria.

We also observed two unintended outcomes of the application of the SPHAR-TI’s model. The first is the detection and reporting of SJS and BG by some participants. SJS is a fatal ADR associated with the use of Nevirapine, a popular antiretroviral drug that constitutes the backbone of first line antiretroviral regimen. This finding confirms that Nigerians are also susceptible to the SJS of Nevirapine as reported in other climes. Further analysis of the submitted ICSRs might reveal other life threatening ADRs in the Nigerian population, which the National Agency for the Control of AIDS (NACA) need to pay close attention to. The second outcome is the interplay of several factors resulting to the increase in the reporting of ADRs. The model combined at least six factors: training, mobilization of participants with resources (refer to Figures A-D in S2 File), a practical reporting system, monitoring and evaluation and providing feedbacks and effective leadership. There is no gainsaying in concluding that training alone without the other factors could not have yielded the results we have reported. Perhaps, the reason Nigeria and other countries are not able to significantly address the challenge of under-reporting of ADRs despite the abundance of training may be the over-reliance on training alone without the other factors.

We observed a surprised finding in the evaluation (S3 Table). Participants without prior training tended to gain knowledge more than those who have attended pharmacovigilance trainings. The same tendency was observed among the health care workers, with pharmacists and nurses gaining knowledge more than the medical doctors. We do not have a viable reason for this but we suspect that personal commitment and seriousness may have led to the difference.

The model holds an important lesson for sub-Saharan Africa (SSA), which has the largest public health programs treating millions of people with HIV, Tuberculosis and Malaria. Currently, over 20 million people must be placed on antiretroviral drugs according to the new WHO treatment guidelines for HIV [60]. The risk of “antiretroviral therapy associated ADRs” is expected to be higher in this region than any other region in the world. Evidence from studies conducted in developed countries where antiretroviral therapy has been offered for many years have reported a rise of cardiometabolic disorders like type 2 diabetis mellitus and cardiovascular disease [61–65], which have long been associated with the antiretroviral medications. In the Drug Resistant Tuberculosis public health program, hearing loss associated with the use of the injectable aminiglycosides (Amikacin and Kanamycin) is a major clinical challenge [66–67] and yet, thousands of patients are using these drugs in the communities. As sub-Saharan African countries continue to scale-up public health treatment programs in a wider global effort to end HIV and Tuberculosis, the prevalence of ADRs will continue to increase, justifying the need for the training of healthcare providers for ADRs reporting.

Another factor that favors the training of healthcare providers for ADR reporting, which justifies the use of the SPHAR-TI model, is the acute shortage of healthcare workers in Africa. The “Brain Drain” report by Rebecca Coombes showed that the number of healthcare workers in many African countries is shrinking [68]. Ghana with a total population of 20 million people has only 1500 medical doctors and more than two-third of young Ghanaian doctors leave the country within three years of graduation. In Mozambique, a nation of similar size with Ghana, there are only 500 medical doctors [68]. Malawi has a worse situation; there are 12 million people but only 350 medical doctors are available to cater for all the health needs including the reporting of ADRs [68]. Nigeria appears to have the highest density of healthcare workers in Africa [37–54] but the large population size and the lack of capacity for reporting ADRs are major constrains. However, the training of healthcare workers as has been shown in several studies can improve the reporting of ADRs. The WHO in its 2013 report on “research for universal health coverage”, highlighted the need for training of healthcare workers in public health programs close to the supply and demand side of health services [69]. The structured pharmacovigilance capacity building model that we have evaluated addresses this gap in response to the WHO recommendation.

An important piece of information the SPHAR-TI model has demonstrated is that short training alone is not sufficient to stem the tide of under-reporting of ADRs. In fact, most developing countries, including Nigeria, provide trainings to healthcare workers to boost the reporting of ADRs but the crisis of under-reporting is not going away. What may be lacking are some of the factors the SPHAR-TI model seems to illustrate, which include: poor mobilization of healthcare providers, a weak monitoring and evaluation with complete absence of feedback mechanisms when ICSRs are submitted to central regulatory authorities, lack of a clear and practical means of submitting ICSRs, lack of private-public collaboration, weak leadership and low motivation of the workforce. If all these factors are combined appropriately, the reporting of ADRs could significantly increase.

The model has some limitations that need to be considered alongside the positive outcomes. The participants were practicing doctors, nurses and pharmacists with some experience in the pharmacotherapy of AIDS, tuberculosis and malaria. This knowledge may have contributed to the knowledge gained through the SPHAR-TI training. This argument may however not hold true because the medical doctors expected to have the highest level of knowledge and should have demonstrated higher scores in the post-test compared to the pharmacists and nurses actually scored less. The age of the participants is another factor; majority of the participants were mid-career professionals occupying lower positions of responsibilities and were likely to be less busy and quick learners. Elderly people with many social and professional responsibilities and perhaps with a “slow to learn” disposition would probably have performed poorly. But again, the results in S2 Table demonstrate that participant over 40 years scored higher marks than the younger people within the age bracket of 30–39 years. Overall, the long term impact of the model need to be assessed; our findings in this study are only limited to the period of evaluation, which is between 10 to 12 months. However, despite these limitations, the SPHARTI model has provided an option for improving the reporting of ADRs in resource limited settings.

We are recommending the use of the SPHAR-TI’s model to minimize the worrisome under-reporting of ADRs in the developing world. As stated earlier, under-reporting of ADRs prevents drug safety monitoring and regulation, which adds to the disease burden and mortality. Nigeria and other developing countries may not be able to absorb additional health challenges caused by ADRs as these countries are already overstretched by communicable and non-communicable diseases. The SPHAR-TI model may be an effective approach that would complement existing models of ADRs reporting in Africa and elsewhere.

## Conclusion

The systematic and output driven training and follow-up of healthcare providers had a positive impact on the reporting of ADRs. The SPHAR-TI principles effectively contributed to the success of the model and are recommended to institutions or organizations providing pharmacovigilance services in Africa and other regions with similar settings.

## Supporting information

S1 File **Figure A:** Adverse Drug Reactions Form; **Figure B**: Letter of appreciation in respect of collaborative work to increase adverse drug reactions reporting in Nigeria.
(DOCX)

S2 File Protocol of the Structured Pharmacovigilance and Training Initiative. (DOCX)

S1 Table Participants’ characteristics, N = 55. (DOCX)

S2 Table Association between post test scores and participants’ characteristics. (DOCX)

S3 Table The outcomes of the SPHAR-TI model. (DOCX)

S1 Dataset S1 DATA.xls. (XLSX)

S2 Dataset S2 DATA.xls. (XLSX)

S3 Dataset S3 DATA.xls. (XLSX)

S4 Dataset S4 DATA.xls. (XLSX)

S5 Dataset S5 DATA.xls. (XLSX)
