# Supplementary material for: Addressing the under-reporting of adverse drug reactions in public health programs controlling HIV/AIDS, Tuberculosis and Malaria: A prospective cohort study
Source: PLoS One. 2018 Aug 22;13(8):e0200810. doi: 10.1371/journal.pone.0200810 (PMC6104922; doi:10.1371/journal.pone.0200810)
Supplement: S1 File — Figure A: Adverse Drug Reactions Form; Figure B: Letter of appreciation in respect of collaborative work to increase adverse drug reactions reporting in Nigeria. (DOCX) [file pone.0200810.s001.docx]

**S1 FILE**

**Figure A**


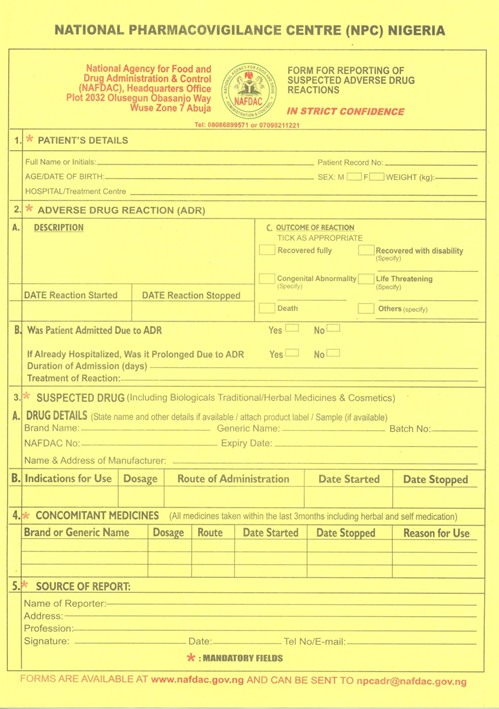


**Source:** The National Agency for Food and Drug Administration and Control, available at: www.nafdac.gov.ng

Figure B

“Reprinted from “Reference NAFDAC/PV/PMS/GC/10/111/360” under a CC BY license, with permission from the “National Agency for Food and Drug Administration and Control”, original copyright, 9^th^ January, 2017”
