# Supplementary material for: Addressing the under-reporting of adverse drug reactions in public health programs controlling HIV/AIDS, Tuberculosis and Malaria: A prospective cohort study
Source: PLoS One. 2018 Aug 22;13(8):e0200810. doi: 10.1371/journal.pone.0200810 (PMC6104922; doi:10.1371/journal.pone.0200810)
Supplement: S2 File — (DOCX) [file pone.0200810.s002.docx]

**S2 FILE**

**Protocol of the Structured Pharmacovigilance and Training Initiative**

**Background**

The Structured Pharmacovigilance and Training Initiative (SPHAR-TI) was designed to improve drug safety through the accurate reporting of adverse drug reactions (ADRs) in public health programs treating the Human Immunodeficiency Virus (HIV), Tuberculosis and Malaria.

ADRs are a global concern because they increase the burden of disease and mortality according to many studies [1-7]. The immediate remedy is the persistent reporting of suspected ADRs to regulatory authorities for drug safety surveillance [8]. However, the under-reporting of ADRs to regional drug regulatory bodies and the international drug monitoring center is a source of global concern [9, 10]. In Nigeria, the high rate of under-reporting of ADRs has been reported in several studies [11-14]. The National Agency for Food and Drug Administration and Control (NAFDAC) – the government agency responsible for drug safety in Nigeria, reported that only 16,500 completed ADR forms were submitted to NAFDAC out of 80,000 uncompleted ADR forms distributed nation-wide over a period of 12 years [15]. A review of the NAFDAC database also showed that a high proportion of the submitted reports were of poor quality. For instance, of the 10,099 Individual Case Safety Reports (ICSRs) sent to the Uppsala Monitoring Center Vigibase, only 94 reports were rated as 100% complete. Overall, over 4,000 reports were quarantined due to poor quality. Judging by these reports, we conclude that the National Pharmacovigilance System the Nigerian government instituted when the country joined the international drug monitoring scheme in 2004, is grossly under-performing, and a complementary model is required. The complimentary model is the SPHAR-TI model, which was funded by the TDR, the Special Programme for Research and Training in Tropical Diseases co-sponsored by UNICEF, UNDP, the World Bank and WHO (Grant number B40396).

**Design and conceptual frame work**

Before developing the SPHARTI model, the Nigerian National Pharmacovigilance System was analyzed to identify the gaps to be strengthened with the model. Six major gaps were identified: limited capacity and skills to detect and report ADRs, inadequate mobilization, a weak monitoring, evaluation and feedback mechanism, absence of a practical means of submitting ICSRs, weak leadership and absence of a collaborative framework for public- private collaboration. Some of these gaps have also been reported in peer-reviewed studies [11-14] and in several technical reports of NAFDAC.

SPHAR-TI was a 12 month modular course, divided into two phases – one week of intensive workshop and nine months of monitoring and evaluating the performance of the participants. The model was based on the World Health Organization (WHO) accredited Structured Operational Research and Training Initiative (SORT-IT) model, designed for the training of public health program staff in operational research in low to medium income countries [16, 17]. The primary aim of SPHAR-TI was to increase the accurate reporting of ADRs and promotes drug safety surveillance in the HIV/AIDS, Tuberculosis and Malaria control programs. There were two major objectives:

1. To train healthcare providers to offer pharmacovigilance services in the public health control of HIV/AIDS, Tuberculosis and Malaria.
2. To develop a pharmacovigilance training model based on practical principles that could be deployed to increase the reporting of ADRs in public health programs.

**The Implementation of SPHAR-TI**

Six distinct but inter-related activities, referred to as the SPHAR-TI’s principles, were implemented. These are: a training workshop; participants’ mobilization; monitoring and evaluating and providing feedback; setting up a reporting system; leadership and collaboration with the government. These principles are briefly described below:

**Training workshop**

The course was publicized in two mainstream national Newspapers with widespread coverage of the country. Participants working in HIV, TB and Malaria public health programmes with no or minimal knowledge in pharmacovigilance were selected from the six geopolitical regions based on strict selection criteria (Table A). A six day intensive workshop with the main objective of building participants’ capacity to accurately detect and report ADRs using the official reporting form was conducted in Abuja. .

The HIV, TB and Malaria public health programs were prioritized because a large number of patients are treated through these programs and are inadvertently exposed to the risks of ADRs. Some studies have reported several incidences of ADRs in patients undergoing treatment through these programs [18-22].

A competent faculty with supportive staff was recruited for the course. There were 12 core professionals including, a Professor of Clinical Pharmacology who was the Chairman of Nigeria’s National Drug Safety Advisory Committee and a Member of the WHO Advisory Committee on the Safety of Medicinal Products and Vaccines, a Malariologist who has served as a National Professional Officer for the Malaria Unit of WHO, an Associate Professor of Medicine who is also an expert HIV/AIDS clinician in the Jos University Teaching Hospital, expert TB clinicians who served at the WHO at different capacities. The list of facilitators also included a Clinical and Public Health Pharmacist and experts in drug regulation from the NAFDAC.

The faculty developed the training manual and the ADR charts and also facilitated the delivery of the content during the workshop as well as reviewed the submitted reports during monitoring and evaluation phase of the course. The major topics covered are presented in Table B.

Didactic lectures, break-out group discussions and plenaries were the main methods of content delivery.

A structured questionnaire – designed to cover the main topics of the course, was administered to assess participants’ knowledge before the training (pre-test). At the end of the workshop, the same questionnaire was re-administered (post-test) to assess the knowledge gained. Participants were not pre-informed they would repeat the test and the results of the pre-test were not disclosed to them. This way, participants went through the training without feelings of excitement or depression for passing or failing the pre-test. The baseline score for assessing the performance in both tests was 45%.

**Participants’ mobilization**

The training was a fully funded residential workshop at no cost to the participants. All training materials were provided free of costs to the participants. A modest allowance was provided to cover out of pocket expenses during the workshop. No financial inducement was otherwise provided. The resources that were provided to the participants to aid the training are:

1. A comprehensive training manual **(Figure A)** [23] covering the pharmacotherapy of HIV, Tuberculosis and Malaria, the ADRs associated with anti-retroviral, anti-tuberculosis and anti-malaria drugs, Pharmacovigilance and the ADR reporting system in the country.
2. ADR Charts for HIV/AIDS, Tuberculosis and Malaria **(Figures B- D)** [24-26].

**Monitoring and evaluating performance and providing feedbacks**

Participants’ performance was monitored and evaluated (M and E) for a period of nine months after the workshop. The purpose of the M and E was to provide further guidance on the accurate completion of the ADR forms. Participants were trained to correctly complete the ADR Forms to minimize poor quality ICSRs. Participants also received regular guidance and feedback on the completion of the ADR forms. The participants met the following milestones:

- Submitted ICSRs (completed ADR Forms) to NAFDAC using a dedicated e-mail platform.
- Shared the ICSRs with the Pharmacovigilance Committees or other stakeholders in the participants’ health facility.
- Conducted step down trainings to optimize pharmacovigilance service at the health facility.
- Collaborated with other healthcare workers to set up pharmacovigilance committees.

The SPHAR-TI’s faculty analyzed the submitted ICSRs and gave feedback through the group email platform. Participants who had problems treating any reported ADRs shared their issues in a discussion group moderated by the faculty.

**Establishing an online system for reporting ADRs to NAFDAC**

Internet web-based system was designed for submitting the ADRs to NAFDAC. Completed ADR forms (ICSRs) were scanned and sent to NAFDAC through the online system. NAFDAC extracted information from the online system into the VigiBase. We monitored the timeliness in the submission and completeness of the ICSRs.

**Collaborating with the Government of Nigeria**

The development and implementation of the model involved partnership with the NAFDAC, the academia and health institutions. Specific activities to be executed by each partner in the partnership were identified (Table C):

**Leadership**

Leadership was provided to the participants through the following activities: casting a clear vision and establishing milestones to be met, motivation, guidance, monitoring and evaluating performance and providing feedbacks through constant communication. By maintaining communication and providing feedbacks when ICSRs were submitted by the participants, a team spirit was built. We also maintained a register of the participants after the workshop. Participants have been encouraged to continue reporting ADRs to NAFDAC through the same online system.

**References**

1. Beijer HJ, Blaey CJ. Hospitalisations caused by adverse drug reactions (ADR): a meta-analysis of observational studies. PharmWorld Sci. 2002; 24(2):46–54.
2. Einarson TR. Drug-related hospital admissions. Ann Pharmacother. 1993; 27(7–8):832–40.
3. Kongkaew C, Noyce PR, Ashcroft DM. Hospital admissions associated with adverse drug reactions: a systematic review of prospective observational studies. Ann Pharmacother. 2008; 42(7):1017–25.
4. Lazarou J, Pomeranz BH, Corey PN. Incidence of adverse drug reactions in hospitalized patients: a meta-analysis of prospective studies. JAMA. 1998; 279(15):1200.
5. Pirmohamed M, James S, Meakin S, Green C, Scott AK, Walley TJ, et al. Adverse drug reactions as cause of admission to hospital: prospective analysis of 18 820 patients. BMJ. 2004; 329(7456):15–9.
6. Paul E, End-Rodrigues T, Thylen P, BergmanU. Adverse drug reactions a common cause of hospitalization of the elderly. A clinical retrospective study. Lakartidningen. 2008; 105(35):2338–42.
7. Baker GR, Norton PG, Flinto V, Blais R, Brown A, Cox J, et al. The Canadian adverse events study: the incidence of adverse events among hospital patients in Canada. CMAJ. 2004; 170(11):1678–86.
8. Wysowski DK, Swartz L . Adverse drug event surveillance and drug withdrawals in the United States, 1969-2002: the importance of reporting suspected reactions. Arch Intern Med. 2005, 165: 1363-1369.
9. Lopez-Gonzalez E, Herdeiro MT, Figueiras A. Determinants of under-reporting of adverse drug reactions: a systematic review. Drug Saf. 2009, 32: 19-31.
10. Hazell L, Shakir SA. Under-reporting of adverse drug reactions: a systematic review. Drug Saf; 2006; 29: 385-396.
11. Chinenye JU, Michael OU. Heath workers and hospital patients’ knowledge of pharmacovigilance in Sokoto, North-West, Nigeria. Nig J Pharmac Sci. 2012; 11 (2): 31-40.
12. Fadare JO, Enwere OO, Afolabi AO, Chedi BAZ, Musa A. Knowledge, Attitude and Practice of Adverse Drug Reaction Reporting among Healthcare Workers in a Tertiary Centre in Northern Nigeria. Tropical Journal of Pharmaceutical Research. 2011; 10 (3): 235-242
13. [Ohaju-Obodo JO](http://www.ncbi.nlm.nih.gov/pubmed?term=Ohaju-Obodo%20JO%5BAuthor%5D&cauthor=true&cauthor_uid=20014358), Iribhogbe OI. Extent of pharmacovigilance among resident doctors in Edo and Lagos states of Nigeria. Pharmacoepidemiology Drug Safety. 2010; 19(2):191-5.
14. [Oreagba IA](http://www.ncbi.nlm.nih.gov/pubmed?term=Oreagba%20IA%5BAuthor%5D&cauthor=true&cauthor_uid=20845410), [Ogunleye OJ](http://www.ncbi.nlm.nih.gov/pubmed?term=Ogunleye%20OJ%5BAuthor%5D&cauthor=true&cauthor_uid=20845410), Olayemi SO. The knowledge, perceptions and practice of pharmacovigilance amongst community pharmacists in Lagos state, south west Nigeria. Pharmacoepidemiol Drug Safety. 2011; 20(1):30-5.
15. Speech of the Acting Director of the National Agency for Food and Drug Administration and Control, delivered on the 3^rd^ March, 2016 in Abuja during the occasion of declaring the SPHARTI Workshop opened.
16. Ramsay A, Harries AD, Zachariah R, Bissell K, Hinderaker SG, Edginton M, D. A. Enarson D.A, Satyanarayana S, Kumar AMV, Hoa NB, Tweya H, Reid AJ, Van den Bergh R, Tayler-Smith K, Manzi M, Khogali M, Kizito W, Ali E, Delaunois, Reeder JC. The Structured Operational Research and Training Initiative for public health programs. Public Health Action. 2014, 4(2): 79–84
17. Guillerm N, dar Berger S, Bissell K, Kumar AMV, Ramsay A, Reid AJ, et al. Sustained research capacity after completing a Structured Operational Research and Training (SORT IT) course. Public Health Action. 2016, 6(3): 207-208.
18. Avong YK, Isaakidis P, Hinderaker SG, Van den Bergh R, Ali E, Obembe BO, et al. Doing No Harm? Adverse Events in a Nation-Wide Cohort of Patients with Multidrug-Resistant Tuberculosis in Nigeria. PLoS ONE, 2015 10(3): e0120161.
19. Shehu NY, Ojeh V, Osaigbovo G, Agaba P, Agbaji OA. A 33-year-old patient with humanimmunodeficiency virus on antiretroviral therapy with efavirenz-induced complex partial seizures: a case report. J Med Case Rep, 2016; 10:93.
20. Ogwuche LO, Ojeh VB, London IA, Naima N, Dady C, Finangwai AI, Abah IO, Falang KD, Agaba P, Agbaji OA. Adverse drug reaction reports in an antiretroviral treatment centre in Jos, North Central Nigeria. Br J Pharm Res. 2014; 4(6): 714-721.
21. Agbaji OA, Agaba PA, Ekeh PN, Sule HM, Ojoh RO, Audu E, et al. Efavirenz-induced gynaecomastia in HIV-infected Nigerian men: A report of six cases. Journal of Medicine and Medical Sciences, 2011; 2(11): 1221-1224.
22. [Agu](http://www.picronline.org/searchresult.asp?search=&author=Kenneth+A+Agu&journal=Y&but_search=Search&entries=10&pg=1&s=0) KA, [Oparah](http://www.picronline.org/searchresult.asp?search=&author=Azuka+C+Oparah&journal=Y&but_search=Search&entries=10&pg=1&s=0) AC. Adverse drug reactions to antiretroviral therapy: Results from spontaneous reporting system in Nigeria. Perspectives in Clinical Research. 2013; 4(2): 117-124.
23. 23. National Agency for Food and Drug Administration and Control (NAFDAC) and Institute of Human Virology Nigeria (IHVN). Pharmacovigilance Structured and Training Initiative. File 2. Abuja: Fasuwai Printers. A training manual of Structured Pharmacovigilance and Training Initiative
24. 24. National Agency for Food and Drug Administration and Control (NAFDAC) and Institute of Human Virology Nigeria (IHVN). Adverse effects of antiretroviral medications. File 2. Abuja: Fasuwai Printers. Typical Adverse drug reactions of anti-retroviral drugs
25. 25. National Agency for Food and Drug Administration and Control (NAFDAC) and Institute of Human Virology Nigeria (IHVN). Adverse effects of anti-tuberculosis medications. File 2. Abuja: Fasuwai Printers. Typical Adverse reactions of anti-tuberculosis drugs
26. 26. National Agency for Food and Drug Administration and Control (NAFDAC) and Institute of Human Virology Nigeria (IHVN). Adverse effects of Anti-malaria medications. File 2. Abuja: Fasuwai Printers. Typical Adverse reactions of anti-malaria drugs

**FIGURES AND TABLES**

**Figure A:** SPHARTI Training Manual

**Figure B:** Adverse Drug Reactions Chart for HIV/AIDS treatment

**Figure C**: Adverse Drug Reactions Chart for Tuberculosis treatment

**Figure D**: Adverse Drug Reactions Chart for Malaria treatment

**Table A:** SPHAR-TI’s selection criteria for the participants

| 1 | Full time employee of a public health facility (non-profit health facilities that are opened to the public such as government or faith-based health institutions) providing Anti-retroviral Therapy (ART), Directly Observed Therapy (DOTS) and Roll Back Malaria (RBM) services. |
| --- | --- |
| 2 | Directly involved in the treatment of HIV/AIDs, TB or Malaria under the ART, DOTS and RBM programs. |
| 3 | Early or mid-career men and women with limited administrative responsibilities in the facility. |
| 4 | Pharmacovigilance naïve (i.e. have never been trained on Pharmacovigilance) or only received partial training in pharmacovigilance. |
| 5 | A written letter of commitment to attend the workshop and also complete the monitoring and evaluation. |
| 6 | One reference letter attesting to the candidate’s suitability and potential to complete the course |
| 7 | Graduate or professional qualification or, strong recommendation by the public health program or work-place institution. |
| 8 | Computer literacy and proven competence in the language of instruction (English). |

**Table B:** Training topics and facilitators

| **Main Topic** | **Sub-topics** | **Facilitators** |
| --- | --- | --- |
| Introduction to the Structured Pharmacovigilance and Training Initiative | 1. Understanding the SPHAR-TI Model | Principal investigator of the grant |
| Introduction to pharmacovigilance and pharmacotherapy of HIV/AIDS, Tuberculosis and Malaria | 1. Basics of Pharmacovigilance 2. Pharmacotherapy of HIV/AIDS 3. Pharmacotherapy of Tuberculosis and Drug Resistant Tuberculosis 4. Pharmacotherapy of TB/HIV and DRTB/HIV co-infection 5. Pharmacotherapy of Malaria | A professor of Clinical Pharmacology, a TB physician and clinical pharmacists. |
| Adverse drug reactions of anti-retroviral, anti-tuberculosis and anti-malaria drugs | 1. ADRs and Common Reactions and Common Drug Interactions of anti-retroviral medications 2. ADRs and Common Reactions and Common Drug Interactions of anti-tuberculosis (1^st^ line, 2^nd^ line TB and XDRT) medications 3. ADRs of anti-malaria drug | HIV physician and professor of medicine, DRTB physician and a consultant Malariologist |
| Reporting of adverse drug reactions | 1. Reporting ADRs within Pharmacovigilance Committees in Health Facilities 2. The Pharmacovigilance System in Nigeria- Coordinating Role of the National Pharmacovigilance Center 3. Quality of reports on the NPC NAFDAC Database-Trend Analysis 4. The Individual Case Safety Report (Yellow Form): Structure & how to complete it 5. The Vigigrade system for measuring completeness score 6. More practice in completing the yellow form (Case Studies) 7. Challenges of Effective Communication in Nigerian Pharmacovigilance system 8. Transmission & Management of ICSRs in the Nigerian Pharmacovigilance system | Drug regulatory experts and pharmacists |

**Table C:** The role of partners in the partnership

| **Partner** | **Role** |
| --- | --- |
| Institute of Human Virology, Nigeria | Lead and provision of resources |
| Institute of Human Virology, Nigeria | Quality Control of completed ADR forms |
| National Agency for Food and Drug Administration and Control | Quality of training (consistency with National Pharmacovigilance Policy) |
| National Agency for Food and Drug Administration and Control | Provided ADR forms |
| Academia | Faculty for training and mentoring training |
| Academia | Development of ADRs Chart and training manual |
| Health Institutions | Provided participants for the course |
| Health Institutions | Conducted step-down trainings and utilized training resources to improve ADR reporting |
