# Supplementary material for: Addressing the under-reporting of adverse drug reactions in public health programs controlling HIV/AIDS, Tuberculosis and Malaria: A prospective cohort study
Source: PLoS One. 2018 Aug 22;13(8):e0200810. doi: 10.1371/journal.pone.0200810 (PMC6104922; doi:10.1371/journal.pone.0200810)
Supplement: S1 Table — (DOCX) [file pone.0200810.s003.docx]

**S1 Table**

| **Characteristics** | **N (%)** |
| --- | --- |
| **Age**  <30 years  30-39 years  40+ years | 5 (9.1%)  29 (52.7%)  21 (38.2%) |
| **Gender**  Female  Male | 24 (43.6%)  31 (56.4%) |
| **Profession**  Doctors  Pharmacists  Nurses | 11 (20.0%)  34 (61.8%)  10 (18.2%) |
| **Disease area**  HIV/AIDS  Tuberculosis  Malaria | 39 (70.9%)  10 (18.2%)  6 (10.9%) |
| **Previous training**  No  Yes | 39 (70.9%)  16 (29.1%) |
| **Geopolitical Region**  North-Central  North-West  North-East  South-West  South-East  South-South | 16 (29.1%)  3 (5.6%)  7 (12.7%)  25 (45.6%)  2 (3.6%)  2 (3.6%) |
