# Supplementary material for: Addressing the under-reporting of adverse drug reactions in public health programs controlling HIV/AIDS, Tuberculosis and Malaria: A prospective cohort study
Source: PLoS One. 2018 Aug 22;13(8):e0200810. doi: 10.1371/journal.pone.0200810 (PMC6104922; doi:10.1371/journal.pone.0200810)
Supplement: S2 Table — (DOCX) [file pone.0200810.s004.docx]

S2 Table

| Characteristics | N (%) | Difference score  Mean (SD) | Pvalue |
| --- | --- | --- | --- |
| Age  <30 years  30-39 years  40+ years | 5 (9.1%)  29 (52.7%)  21 (38.2%) | 7.0 (2.7)  6.0 (7.3)  9.4 (7.0) | 0.16 |
| Gender  Female  Male | 24 (43.6%)  31 (56.4%) | 7.5 (7.7)  7.3 (6.5) | 0.96 |
| Profession  Doctors  Pharmacists  Nurses | 11 (20.0%)  34 (61.8%)  10 (18.2%) | 3.6 (4.7)  8.5 (7.4)  7.6 (6.4) | 0.11 |
| Disease area  HIV/AIDS  Tuberculosis  Malaria | 39 (70.9%)  10 (18.2%)  6 (10.9%) | 8.2 (6.9)  3.0 (4.5)  9.3 (8.8) | 0.09 |
| Previous training  No  Yes | 39 (70.9%)  16 (29.1%) | 6.5 (6.5)  9.5 (7.8) | 0.21 |
|  |  |  |  |
