# Supplementary material for: Addressing the under-reporting of adverse drug reactions in public health programs controlling HIV/AIDS, Tuberculosis and Malaria: A prospective cohort study
Source: PLoS One. 2018 Aug 22;13(8):e0200810. doi: 10.1371/journal.pone.0200810 (PMC6104922; doi:10.1371/journal.pone.0200810)
Supplement: S3 Table — (DOCX) [file pone.0200810.s005.docx]

**S3 Table**

| **DESCRIPTION** | **Number (P-value)** |
| --- | --- |
| PARTICIPANTS TRAINED^1^ : Participants trained at workshop vs Healthcare workers trained by participants after the workshop | 55 vs 2937 |
| KNOWLEDGE GAIN^2^ : Mean pre vs post test scores | 20.4 vs 27.8 (P value< 0.001) |
| ADRs REPORTED^3^ : ADR reported by SPHAR-TI trained participants vs ADR reported by Healthcare providers in the general population) | 3000 ICSRs vs 805 ICSRs  (percentage incresease in ADR reporting =273%) |
| PHARMACOVIGILANCE COMMITTEES^4^ : Pharmacovigilance Committees activated by SPHAR-TI participants before the workshop vs Pharmacovigilance committees activated by participants after the workshop | 0 vs 46 |

**NOTES:** 1 = The number of participants trained at the workshop are compared with the number of healthcare workers trained by the participants after the workshop (55 vs 2937). **2** = The mean pre and post test scores (20.4 vs 27.8) are compared; the difference between the two scores is statistically significant suggesting knowledge gained. **3** = The number of ICSRs submitted to the National Agency for Food and Drug Administration (NAFDAC) by the SPHAR-TI trained participants in seven months are compared with the proportion of ICSRs submitted to NAFDAC by all the healthcare providers in Nigeria in seven months (3000 vs 805).

4 = The number of Pharmacovigilance Committees activated by the participants before they were trained is compared with the number they activated after they were trained (0 vs 46).
